# Consultation-liaison psychiatry in a Moroccan university hospital : service requests and patterns.

**DOI:** 10.1192/j.eurpsy.2026.11060

**Published:** 2026-07-31

**Authors:** R. Hayat, S. Boughdadi, S. Karroumi, I. Adali, F. Manoudi

**Affiliations:** Ibn Nafis Hospital, Psychiatry, Mohammed VI university hospital, Marrakech, Morocco

## Abstract

**Introduction:**

Hospitalized patients frequently present with psychiatric symptoms that may go unnoticed, despite their significant impact on recovery and overall care. Consultation-liaison psychiatry addresses this gap by integrating mental health expertise into medical and surgical settings. Understanding patterns of consultation is crucial to improve patient outcomes and guide healthcare teams in recognizing mental health needs early.

**Objectives:**

To examine the prevalence and types of psychiatric disorders encountered in consultation-liaison psychiatry, and to identify the main requesting services and reasons for referral at Mohammed VI university hospital, Marrakech.

**Methods:**

This descriptive study included 110 consultation requests over 10 months (Nov 8, 2024–Sep 8, 2025). Data were collected from the liaison psychiatry register on sociodemographics, hospitalization reason, patient history, requesting service, consultation reason, attitude, management, and outcomes. Descriptive statistics summarized the findings.

**Results:**

A total of 110 psychiatric consultations were analyzed (mean age 39.8 years, 48.6% female). Most patients (66.3%) had no prior psychiatric history, making this their first psychiatric contact. Requests came from 33 medical and surgical services, most frequently the emergency department (14.4%), gynecology-obstetrics (11.7%), and orthopedic traumatology (9%), primarily initiated by caregivers (97%). Main reasons were suicide attempts (19%), sad mood (14.5%), and agitation (7.3%). Patient attitudes were cooperative in 62%; three were confused and one remained unconscious in intensive care. Depressive disorders predominated (28.2%), followed by psychotic disorders (14.4%). An organic work-up was requested in 17.2%, identifying an organic etiology in 10%. Six patients had reactive agitation (e.g., post-MRI). Eleven received psychological support alone. Pharmacological treatment included antidepressants (23.6%) and antipsychotics (11.8%), with six cases using antipsychotics to control agitation for facilitating organic investigations.

**Conclusions:**

Most referrals were prompted by suicide attempts, highlighting psychiatric emergencies as the main driver of liaison consultations, partly explaining the low overall rate of requests outside urgent situations. Reactive agitation in some cases could have been prevented with better communication, emphasizing the need for improved psychosocial approaches in medical and surgical wards. The identification of organic causes in a subset of patients underscores the importance of systematically investigating medical etiologies during psychiatric consultations. Notably, 66.3% of patients had no prior psychiatric contact, underscoring the key role of liaison psychiatry in early detection. These findings highlight the importance of raising awareness among healthcare teams about psychiatric screening to improve overall patient care.

**Disclosure of Interest:**

None Declared

